# 3-Deoxyglucosone Induces Glucagon-Like Peptide-1 Secretion from STC-1 Cells via Upregulating Sweet Taste Receptor Expression under Basal Conditions

**DOI:** 10.1155/2019/4959646

**Published:** 2019-10-23

**Authors:** Xiudao Song, Fei Wang, Heng Xu, Guoqiang Liang, Liang Zhou, Lurong Zhang, Fei Huang, Guorong Jiang

**Affiliations:** ^1^Clinical Pharmaceutical Laboratory of Traditional Chinese Medicine, Suzhou TCM Hospital Affiliated to Nanjing University of Chinese Medicine, Suzhou 215009, Jiangsu, China; ^2^Clinical Pharmaceutical Laboratory of Traditional Chinese Medicine, Suzhou Academy of Wumen Chinese Medicine, Suzhou 215009, Jiangsu, China; ^3^Department of Endocrinology, Suzhou TCM Hospital Affiliated to Nanjing University of Chinese Medicine, Suzhou 215009, Jiangsu, China

## Abstract

3-Deoxyglucosone (3DG) is derived from D-glucose during food processing and storage and under physiological conditions. We reported that glucagon-like peptide-1 (GLP-1) secretion in response to an oral glucose load *in vivo* and high-glucose stimulation *in vitro* was decreased by acute 3DG administration. In this study, we determined the acute effect of 3DG on GLP-1 secretion under basal conditions and investigated the possible mechanisms. Normal fasting rats were given a single acute intragastric administration of 50 mg/kg 3DG. Plasma basal GLP-1 levels and duodenum 3DG content and sweet taste receptor expression were measured. STC-1 cells were acutely exposed to 3DG (80, 300, and 1000 ng/ml) for 1 h under basal conditions (5.6 mM glucose), and GLP-1 secretion, intracellular concentrations of cyclic adenosine monophosphate (cAMP) and Ca^2+^, and molecular expression of STR signaling pathway were measured. Under the fasted state, plasma GLP-1 levels, duodenum 3DG content, and duodenum STR expression were elevated in 3DG-treated rats. GLP-1 secretion was increased in 3DG-treated cells under either 5.6 mM glucose or glucose-free conditions. 3DG-induced acute GLP-1 secretion from STC-1 cells under 5.6 mM glucose was inhibited in the presence of the STR inhibitor lactisole, which was consistent with the observation under glucose-free conditions. Moreover, acute exposure to 3DG increased the protein expression of TAS1R2 and TAS1R3 under either 5.6 mM glucose or glucose-free conditions, with affecting other components of STR signaling pathway, including the upregulation of transient receptor potential channel type M5 TRPM5 and the increment of intracellular Ca^2+^ concentration. In summary, the glucose-free condition was used to first demonstrate the involvement of STR in 3DG-induced acute GLP-1 secretion. These results first showed that acute 3DG administration induces basal GLP-1 secretion in part through upregulation of STR expression.

## 1. Introduction

Glucagon-like peptide-1 (GLP-1), an incretin hormone secreted from intestinal enteroendocrine L-cells by nutrients, has a broad role in glucose homeostasis, in great part through potentiation of glucose-dependent insulin secretion, enhancement of *β*-cell growth and survival, inhibition of glucagon release, and suppression of food intake [1]. Glucose has been reported to induce GLP-1 secretion from mouse jejunal and ileum explants, and mouse (GLUTag and STC-1) and human (NCI-H716) enteroendocrine cell lines [2–5]. Glucose is a natural ligand for the sweet taste receptors (STRs), a heterodimeric G-protein-coupled receptor consisting of two subunits (TAS1R2/TAS1R3) [6]. In TAS1R2^−/−^ or TAS1R3^−/−^ mice, glucose-stimulated GLP-1 secretion is almost or fully abolished [3, 4]. Additionally, electrogenic glucose transport sodium-glucose transporter 1 (SGLT1) and facilitative glucose transporter 2 (GLUT2) are also implicated in glucose-triggered GLP-1 secretion [7, 8]. Taken together, STR activation synergizes with SGLT1 and GLUT2 to involve in glucose-stimulated GLP-1 secretion.

Reactive 1,2-dicarbonyl compounds are generated from carbohydrates during food processing and storage and under physiological conditions [9, 10]. Formation and degradation reactions of 1,2-dicarbonyl compounds that participate in color and aroma form are closely connected to the palatability of food [11]. One of the most abundant 1,2-dicarbonyl compounds in food is 3-deoxyglucosone (3DG) [12]. High 3DG contents were measured in fruit juices (up to 410 mg/L), balsamic vinegar (up to 2622 mg/L), honey (10 mmol/kg), and bakery products such as cookies (up to 385 mg/kg) [12]. In certain diseases such as diabetes and uremia, plasma concentrations of 3DG are elevated [13]. The clinical significance of 3DG lies in the fact that it reacts with and modifies certain proteins to form advanced glycation end products that may lead to a significant impairment of protein function [14]. Our recent results from animal studies and clinical research have revealed that 3DG is an independent factor associated with the development of prediabetes [15–19]. Since the absorption rate of individual dietary 3DG is low [16, 20], 3DG may mainly influence the intestinal tissue. In agreement, our *in vivo* studies in rats identified a role of intestinal deposition of 3DG by a 2-week administration of exogenous 3DG in reducing basal and oral glucose-induced GLP-1 secretion [16]. Interestingly, our previous study found that acute 3DG administration by gastric gavage resulted in the impairment in oral glucose-stimulated GLP-1 secretion in rats [17]. Furthermore, acute exposure of STC-1 cells to 3DG in the presence of high glucose (25 mM) for one hour is able to downregulate the protein expression level of STR, thus decreasing STR-mediated GLP-1 secretion [21]. It is unknown, however, whether acute exposure to 3DG may impair GLP-1 secretion under basal conditions.

In the present study, we investigated the acute effect of 3DG on basal GLP-1 secretion *in vivo* and *in vitro*. 3DG content and STR protein expression in the duodenum of 3DG-treated rats were measured. STR expression levels and its downstream components, including Ca^2+^, cyclic adenosine monophosphate (cAMP), and TRPM5, were also measured under basal conditions (5.6 mM glucose) in STC-1 cells incubated with 3DG for one hour. Glucose-free condition serves as a special condition to determine the acute effect of 3DG on GLP-1 secretion, cAMP levels, Ca^2+^ levels, and STR pathway.

## 2. Materials and Methods

### 2.1. Reagents

On the basis of the method of Kato et al. [20], the synthesis of 3DG was performed as previously described [22, 23]. UV, IR, ^1^H-NMR, ^13^C-NMR, MS, and HPLC-ELSD were used to identify the synthetic 3DG [23]. Phloretin, lactisole, and phloridzin dihydrate were purchased from Sigma-Aldrich (St. Louis, MO). Phloridzin dihydrate, phloretin, and lactisole were prepared as a stock solution in DMSO, and the final DMSO concentration was adjusted to 0.05%.

### 2.2. Animals and STC-1 Cell Culture

Sprague Dawley (SD) rats (11 weeks old) from JOINN Laboratories (Suzhou, China) were treated according to the Guide for the Care and Use of Laboratory Animals published in 2011 by the National Institutes of Health (USA). All of the animal protocols were approved by the Local Ethics Committee of Animal Experiments of Suzhou TCM Hospital Affiliated to the Nanjing University of Chinese Medicine. SD rats were allowed free access to water and a standard rat diet (Shuangshi Laboratory Animal Feed Science Co. Ltd, Suzhou, China). After one week of acclimatization, the SD rats were randomly divided into control and 3DG groups (each consisting of six animals). The SD rats fasted overnight before the experiments. Murine STC-1 cells, an intestinal enteroendocrine cell line, were originally purchased from Cell Bank of the Chinese Academy of Sciences (Shanghai, China). Cells were cultured in Dulbecco's modified Eagle's medium (DMEM; Gibco; Thermo Fisher Scientific, Inc., Waltham, MA, USA) containing 25 mM glucose supplemented with 10% heat-inactivated fetal bovine serum (FBS; Zhejiang Tianhang Biological Technology Co., Huzhou, China), 100 IU/ml penicillin, and 100 ng/ml streptomycin at 37°C in a humidified atmosphere containing 5% CO_2_. The cells were grown to 70–80% confluence for the experiments.

### 2.3. Assay of Extracellular GLP-1

STC-1 cells were seeded into 24-well plates at a density of 2 × 10^5^ cells/well for 48 h. The cells were washed three times with phosphate-buffered saline (PBS) and then incubated with glucose-free DMEM (Gibco; Life Technologies Co., Grand Island, NY14072, USA). After 3 h, the medium was subsequently removed, and the cells were incubated with the indicated reagents for 1 h (see figures) under glucose-free (0 mM) DMEM or low-glucose (5.6 mM) DMEM conditions according to the method of Eiki et al. [24]. After the incubation, the medium was collected and centrifuged at 12 000 × *g* for 5 min at 4°C. The GLP-1 concentration (total) in the supernatant was measured using the Multi-Species GLP-1 Total ELISA kit (Millipore, MA, USA).

### 2.4. Assay of cAMP in Cells

STC-1 cells were seeded into six-well plates for 48 h. The cells were washed three times with PBS and then incubated with glucose-free DMEM. After 3 h, the medium was subsequently removed, and the cells were incubated for 1 h with either 0 or 5.6 mM glucose in the presence and absence of 300 ng/ml 3DG. After the incubation, the medium was removed. Then cells were harvested and cell lysates were prepared and stored at −80°C for later use. The cAMP level in cell lysates was assayed by using the Rat cAMP ELISA kit (Shanghai BangYi Bio-Technology Co., Ltd., Shanghai, China).

### 2.5. Determination of Intracellular Ca^2+^ Levels

Ca^2+^ levels in the cells were determined using a fluorescent probe, Fluo-3/AM. STC-1 cells were seeded as described above. The cells were washed three times with PBS and then incubated with glucose-free DMEM. After 3 h, the medium was subsequently removed, and the cells were continued to incubate with for 1 h with either 0 or 5.6 mM glucose in the presence and absence of 300 ng/ml 3DG. The medium was subsequently removed, and the cells were collected and resuspended in 2 ml PBS. Fluo-3/AM (Beyotime, Jiangsu, China) at final concentrations of 2.5 *μ*M was added for 30 min at room temperature. The cells were washed three times with PBS. The intracellular Ca^2+^ was analyzed at the 488/525–530 nm fluorescence ratio by flow cytometry (BD Accuri C6, Ann Arbor, USA) and analyzed by BD software 1.0 (Franklin Lakes, NJ).

### 2.6. Western Blot Analysis

The expression of protein was characterized via western blot analysis. In brief, after 3 hr treatment with glucose-free DMEM, the STC-1 cells were incubated for 1 h with either 0 or 5.6 mM glucose in the presence and absence of 300 ng/ml 3DG. Then cells were harvested and cell lysates were prepared and stored at −80°C for later use. The protein content in the lysates was determined using the BCA Protein Assay Kit (Beyotime, Jiangsu, China). One hour after a single administration of saline (control) or 50 mg/kg 3DG by gastric gavage, the duodenum tissues form fasted rats were isolated. Methods for protein extraction in tissues have been previously described [19]. For western blot analysis, 50 *μ*g of protein from each sample was subjected to electrophoresis and was transferred to membranes. The blots of proteins in polyvinylidene difluoride (PVDF) membranes were incubated with appropriate primary antibody at 4°C overnight. Subsequently, membranes were washed and then incubated with horseradish peroxidase- (HRP-) conjugated secondary antibody for 2 h at room temperature. The blots were detected with chemiluminescence (ECL-Kit, Beyotime, Jiangsu, China) followed by autoradiography. Quantification of protein bands was performed using ImageJ software version 1.42 (National Institutes of Health, Bethesda, MD, USA). The antibodies used for western blot were as follows: anti-STR subunit TAS1R2 (dilution, 1 : 200; Santa Cruz Biotechnology, Santa Cruz, CA), anti-STR subunit TAS1R3 (dilution, 1 : 200; Santa Cruz Biotechnology, Santa Cruz, CA), and anti-TRPM5 (dilution, 1 : 167; Abcam, Cambridge, MA).

### 2.7. Measurement of Plasma GLP-1 and Duodenum 3DG Content

After overnight fasting, saline (control) or 3DG at 50 mg/kg was administered to the rats by gastric gavage. Blood samples from aorta abdominals were collected before (0 min) and after (15, 30, 60, and 120 min) 3DG administration for the measurements of GLP-1 levels. Plasma GLP-1 (total) concentrations were measured using the Multi-Species GLP-1 Total ELISA kit (Millipore, MA, USA). The area under the curve (AUC) (0–120 min) for GLP-1 was calculated for each group of rats. Duodenum tissues after 1 hr administration of 3DG (50 mg/kg) were obtained for the measurement of 3DG contents by high-performance liquid chromatography, as described previously [16].

### 2.8. Statistical Analysis

All of the data shown are representative of at least three independent experiments and are expressed as the mean ± SD. Statistical significance of differences was assessed by Student's *t*-test or ANOVA followed by Dunnett's multiple comparison posttest (GraphPad Prism Software, San Diego, CA, USA). *P* values ≤ 0.05 were considered statistically significant.

## 3. Results

### 3.1. Acute Effects of 3DG on Basal GLP-1 Secretion *In Vivo* and in STC-1 Cells (Figures 1(a)–1(d))

**Figure 1:**
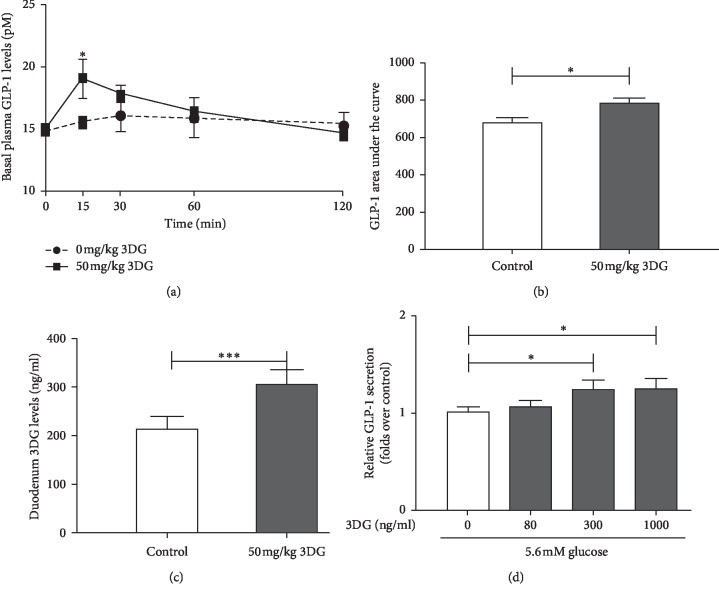
Acute effects of 3DG on basal GLP-1 secretion *in vivo* and in STC-1 cells. Saline (control) or 3DG at 50 mg/kg was administered to the fasted rats by gastric gavage. Blood samples were collected from the aorta abdominals before (0 min) and after (15, 30, 60, and 120 min) 3DG administration. (a) Plasma GLP-1 concentration was measured by ELISA and (b) the area under curves for plasma GLP-1 was evaluated. ^*∗*^*P* < 0.05, compared with the control group at the same point (a), ^*∗*^*P* < 0.05 compared with the control group (b) (*n* = 3). (c) Saline (control) or 3DG at 50 mg/kg was administered to the fasted rats by gastric gavage. After one hour, the duodenum tissue was obtained for the measurement of 3DG content. ^*∗∗∗*^*P* < 0.001 (*n* = 3). (d) STC-1 cells were incubated for 1 h with 5.6 mM glucose in the presence and absence of 3DG. The GLP-1 concentration in the supernatant was measured by ELISA. The data are expressed as a percent of the untreated group. ^*∗*^*P* < 0.05 (*n* = 6).

To examine the acute effect of a single oral administration of 3DG on basal plasma levels of GLP-1, normal fasted rats were given a single acute intragastric administration of 50 mg/kg 3DG that has been chosen in our previous studies [16]. As shown in Figure 1(a), fasting plasma level of GLP-1 at 15 min point in the 3DG-treated group (19.1 ± 1.556 pM) was significantly (*P* < 0.05) higher than the control group (15.57 ± 0.297 pM). Consistently, AUC for 0–120 min of GLP-1 levels was significantly (*P* < 0.05) increased in 3DG-treated rats (780.8 ± 78) compared with the control group (681.2 ± 50.4) (Figure 1(b)). The duodenum 3DG level was significantly increased in the 3DG-treated rat compared with the control (control, 214.25 ± 24.76 ng/ml; 50 mg/kg 3DG, 305.25 ± 29.53 ng/ml; ^*∗∗∗*^*P* < 0.001; Figure 1(c)). Then, we examined whether acute exposure to 3DG could stimulate GLP-1 secretion from STC-1 cells under 5.6 mM glucose conditions. As shown in Figure 1(d), in STC-1 cells after 1 hr exposure to either 300 ng/ml or 1000 ng/ml 3DG, GLP-1 secretion in the presence of 5.6 mM glucose was significantly (*P* < 0.05) increased by 1.23-folds.

### 3.2. Acute Effects of 3DG on GLP-1 Secretion and Intracellular Ca^2+^ and cAMP Levels in STC-1 Cells under Glucose-Free Conditions (Figures 2(a)–2(c))

**Figure 2:**
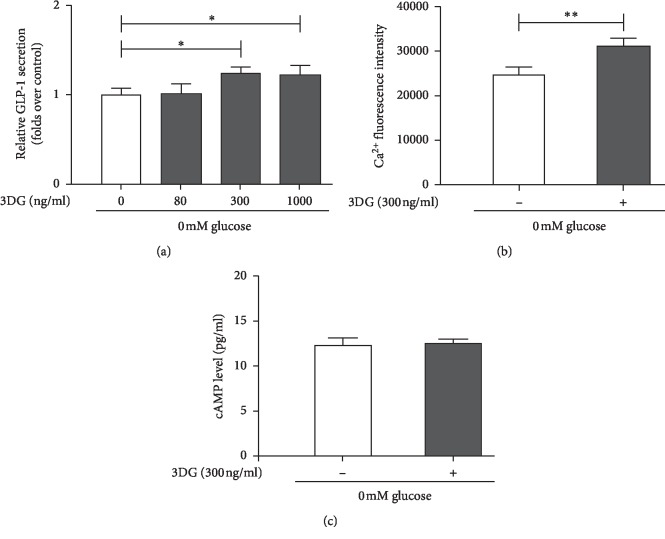
Acute effects of 3DG on GLP-1 secretion and intracellular Ca^2+^ and cAMP levels in STC-1 cells under glucose-free conditions. STC-1 cells were incubated for 1 h with free glucose in the presence and absence of 3DG. (a) The GLP-1 concentration in the supernatant was measured by ELISA. The data were expressed as a percent of the untreated group. ^*∗*^*P* < 0.05 (*n* = 6). (b) The Ca^2+^ concentrations in STC-1 cells were measured at the 488/525–530 nm fluorescence ratio in cells loaded with Fluo-3/AM. ^*∗∗*^*P* < 0.01 (*n* = 6). (c) The intracellular cAMP level was assayed by ELISA (*n* = 6).

To explore whether acute exposure to 3DG for 1 h could directly stimulate GLP-1 secretion from STC-1 cells, the glucose-free condition was chosen to avoid the interference of glucose. As shown in Figure 2(a), GLP-1 secretion from STC-1 cells was markedly (*P* < 0.05) increased by ∼1.23-folds in 300 ng/ml or 1000 ng/ml 3DG-treated group compared to the control group, which is in agreement with the results observed under basal conditions. A lower concentration (80 ng/ml) of 3DG was not effective (Figure 2(a)). We next assessed the acute effects of 300 ng/ml 3DG on intracellular levels of Ca^2+^ and cAMP under glucose-free conditions. Acute administration of 300 ng/ml 3DG for 1 h dramatically (*P* < 0.01) increased fluorescence compared to basal levels (1.26-folds over basal values) (Figure 2(b)), while under glucose-free conditions, acute exposure to 300 ng/ml 3DG did not affect intracellular cAMP levels (Figure 2(c)).

### 3.3. Possible Involvement of STRs, SGLT1, or GLUT2 in 3DG-Induced Acute GLP-1 Secretion in STC-1 Cells under Glucose-Free Conditions (Figures 3(a)–3(c))

**Figure 3:**
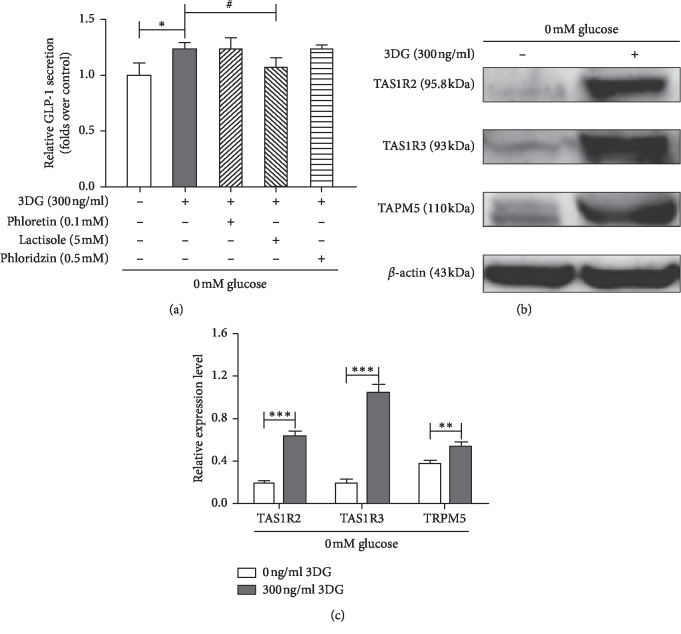
Possible involvement of STRs, SGLT1, or GLUT2 in 3DG-induced acute GLP-1 secretion in STC-1 cells under glucose-free conditions. (a) STC-1 cells were incubated for 1 h with 0 or 300 ng/ml 3DG in the presence and absence of lactisole (5 mM), phloretin (0.1 mM), and phloridzin (0.5 mM) under glucose-free conditions. The GLP-1 concentration in the supernatant was measured by ELISA. The data are expressed as a percent of the untreated group. ^*∗*^*P* < 0.05, ^#^*P* < 0.05 (*n* = 6). (b) STC-1 cells were incubated for 1 h with 0 mM glucose in the presence and absence of 300 ng/ml 3DG. The protein expression of STR subunits (TAS1R2 and TAS1R3) and TRPM5 was measured. (c) The protein levels of TAS1R2, TAS1R3, and TRPM5 were analyzed by ImageJ software. ^*∗∗*^*P* < 0.01, ^*∗∗∗*^*P* < 0.001 (*n* = 3).

To further explore the possible mechanisms by which acute exposure to 3DG directly stimulates GLP-1 secretion, we investigated the involvement of gut glucose sensors liking STRs, SGLT1, and GLUT2 in 3DG-induced acute GLP-1 secretion under glucose-free conditions. Under glucose-free conditions, acute administration of 300 ng/ml 3DG significantly increased GLP-1 secretion (1.24-folds vs. basal values), but in the presence of the STR inhibitor lactisole, 3DG-induced GLP-1 secretion was markedly reduced to 86% (Figure 3(a)), whereas under glucose-free conditions, exposure of STC-1 cells to either the SGLT1 inhibitor phloridzin or the GLUT2 inhibitor phloretin did not affect 3DG-induced acute GLP-1 secretion (Figure 3(a)). Thus, we further investigated the acute effects of 3DG on the protein expression of the STR signaling molecule under glucose-free conditions. STC-1 cells were exposed to 300 ng/ml 3DG for 1 h under glucose-free conditions, and then STR subunits (TAS1R2 and TAS1R3) and their downstream signaling component TRPM5 were measured. As shown in Figures 3(b) and 3(c), treatment with 300 ng/ml 3DG significantly increased the protein expression levels of TAS1R2, TAS1R3, and TRPM5 under glucose-free conditions. Under glucose-free conditions, these results demonstrated the involvement of STRs in 3DG-induced acute GLP-1 secretion from STC-1 cells.

### 3.4. STR Is Involved in 3DG-Induced Acute GLP-1 Secretion in STC-1 Cells and Rats under Basal Conditions (Figures 4(a)–4(d))

**Figure 4:**
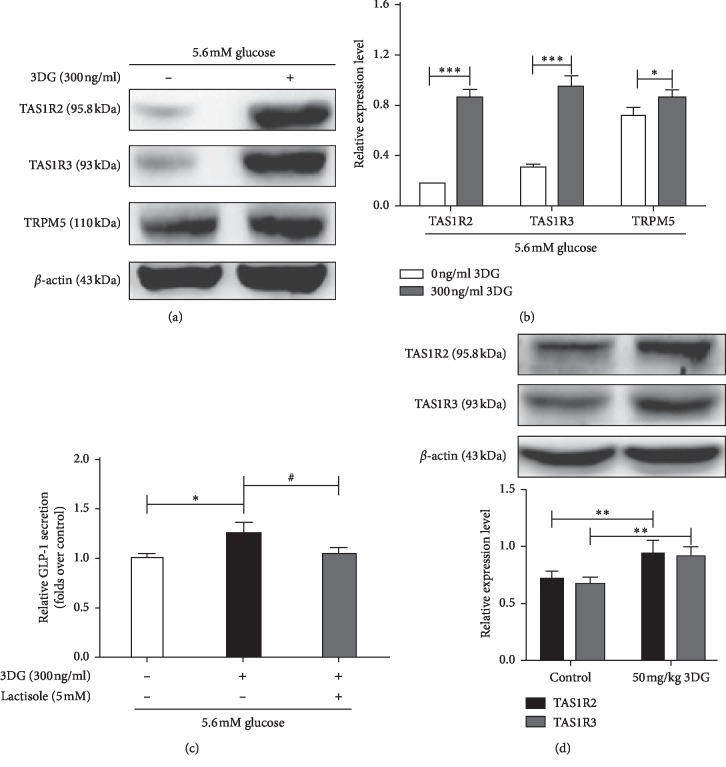
STR is involved in 3DG-induced acute GLP-1 secretion in STC-1 cells and rats under basal conditions. STC-1 cells were incubated with 5.6 mM glucose for 1 h in the presence and absence of 300 ng/ml 3DG. (a) The protein expression levels of TAS1R2, TAS1R3, and TRPM5 were measured by western blot. (b) The protein levels of TAS1R2, TAS1R3, and TRPM5 were analyzed by ImageJ software. ^*∗*^*P* < 0.05, ^*∗∗∗*^*P* < 0.001 (*n* = 3). (c) STC-1 cells were incubated for 1 h with either 0 or 300 ng/ml 3DG in the presence and absence of 5 mM lactisole under 5.6 mM glucose conditions. The GLP-1 concentration in the supernatant was measured by ELISA. The data are expressed as a percent of the untreated group. ^*∗*^*P* < 0.05, ^#^*P* < 0.05 (*n* = 6). (d) Saline (control) or 3DG at 50 mg/kg was administered to the fasted rats by gastric gavage for 1 h. The protein levels of TAS1R2 and TAS1R3 in the duodenum of 3DG-treated rats were measured by western blot and then were analyzed by ImageJ software. ^*∗∗*^*P* < 0.01 (*n* = 3).

We next investigated whether the STR participates in 3DG-induced acute GLP-1 secretion under basal conditions. First, we investigated the acute effects of 3DG on the molecular expression of the STR signaling pathway in STC-1 cells under 5.6 mM glucose conditions. As shown in Figures 4(a) and 4(b), 300 ng/ml 3DG significantly upregulated the protein expression levels of TAS1R2 and TAS1R3 as well as their downstream molecule TRPM5. We then used the STR inhibitor lactisole to investigate the role of STRs in 3DG-induced acute GLP-1 secretion under 5.6 mM glucose conditions. Acute administration of 300 ng/ml 3DG significantly increased GLP-1 secretion (1.25-folds vs. basal values), but in the presence of the STR inhibitor lactisole, 3DG-induced GLP-1 secretion was markedly reduced to 84% (Figure 4(c)). These results indicated the involvement of STRs in 3DG-induced potentiation of low-glucose-stimulated GLP-1 secretion in STC-1 cells. Second, we also investigated whether STR expression is also altered in the duodenum of 3DG-treated rats. As expected, the protein levels of TAS1R2 and TAS1R3 in the duodenum were significantly increased in 50 mg/kg 3DG-treated rats compared to the control group (Figure 4(d)).

### 3.5. Acute Effects of 3DG on Intracellular Ca^2+^ and cAMP Levels in STC-1 Cells under Basal Conditions (Figures 5(a) and 5(b))

**Figure 5:**
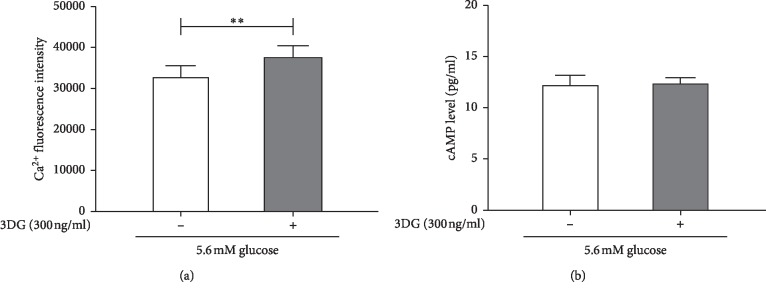
Acute effects of 3DG on intracellular Ca^2+^ and cAMP levels in STC-1 cells under basal conditions. STC-1 cells were incubated for 1 h with 0 mM glucose in the presence and absence of 3DG. (a) The Ca^2+^ concentrations in STC-1 cells were measured at the 488/525–530 nm fluorescence ratio in cells loaded with Fluo-3/AM. ^*∗∗*^*P* < 0.01 (*n* = 6). (b) The intracellular cAMP level was assayed by ELISA (*n* = 6).

To investigate the intracellular signaling systems activated by the STRs, we measured the acute effects of 3DG on intracellular levels of Ca^2+^ and cAMP under basal conditions. As shown in Figure 5(a), acute exposure of STC-1 cells to 300 ng/ml 3DG for 1 h induced a significant (*P* < 0.05) increase in the intracellular Ca^2+^ level in the presence of 5.6 mM glucose (37914.54 ± 2694.47 in the 3DG-treated group vs. 32564.28 ± 3282.29 in the control group), whereas under 5.6 mM glucose conditions, no significant difference was observed in intracellular cAMP levels upon treatment of 300 ng/ml 3DG for 1 h (Figure 5(b)).

## 4. Discussion

Previously, we have reported that an acute administration of 3DG leads to a decrease in glucose-stimulated GLP-1 secretion *in vivo* [17] and *in vitro* [21]. In this study, we examined the effect of an acute administration of 3DG on basal GLP-1 secretion *in vitro* and *in vivo*. Moreover, we investigated the involvement of glucose sensors in the gut to explore the possible mechanisms. Our *in vivo* results showed that basal plasma GLP-1 levels and duodenum 3DG content and STR expression were elevated by a single oral administration of 3DG in rats. Consistently, under 5.6 mM glucose conditions, acute exposure to 3DG stimulated GLP-1 secretion from the enteroendocrine STC-1 cell line. Furthermore, the results observed under glucose-free conditions demonstrated the involvement of STR activation in 3DG-triggered GLP-1 secretion. Importantly, under basal conditions, GLP-1 secretion induced by 3DG in STC-1 cells was inhibited in the presence of the STR inhibitor lactisole. Moreover, acute exposure to 3DG upregulated the STR expression as well as its downstream molecule TRPM5. These results reveal that the stimulatory effect of an acute administration of 3DG on GLP-1 secretion under basal conditions should be attributed to STR activation.

The involvement of STRs in GLP-1 secretion caused by natural sweeteners (sucrose, glucose, and fructose), artificial sweeteners, and glucose analogue (3-*O*-methylglucose) has been demonstrated by the pharmacological and genetic interference with STRs [2, 4, 25]. Under glucose-free conditions, our results showed that an acute administration of 3DG, a dietary ingredient, directly stimulates GLP-1 secretion. Furthermore, acute exposure to 3DG upregulated the STR expression. It has been documented that STR activation mobilizes Ca^2+^ from intracellular stores by the activation of phospholipase C and inositol triphosphate [26]. The increase in intracellular Ca^2+^ opens the TRPM5, leading to Na^+^ influx, depolarization of the cell, and eventual hormone secretion [27]. Indeed, the elevation of intracellular Ca^2+^ and the upregulation of TRPM5 expression were observed in 3DG-treated STC-1 cells with STR activation. These results indicated that 3DG-induced acute GLP-1 secretion under glucose-free conditions is associated with STR activation. With this in mind, STR inhibitor is a useful *in vitro* tool to further evaluate the role of STR activation in 3DG-induced acute GLP-1 secretion. Although lactisole is a human-specific STR inhibitor, some recent reports have suggested that it may be useful in assessing the role of STRs in rodents [28, 29]. In this study, under glucose-free conditions, the STR inhibitor lactisole but not the SGLT1 inhibitor phloridzin or the GLUT2 inhibitor phloretin inhibited 3DG-induced acute GLP-1 secretion, which further indicated the involvement of STR activation in the 3DG-induced acute GLP-1 secretion. Besides, the results also suggest that lactisole is a useful pharmacological tool to assess the function and physiological role of STRs in STC-1 cells. Collectively, under glucose-free conditions, acute 3DG treatment directly activated STR to trigger GLP-1 secretion.

Based on the present results observed under glucose-free conditions, it is reasonable to speculate that 3DG may be a STR ligand. If so, competitive inhibition between 3DG and high glucose may emerge. Interestingly, we previously reported that acute exposure to 3DG reduces GLP-1 secretion by downregulating the STR expression under high-glucose conditions [21]. The above results could be explained by the idea that 3DG treatment may partially occupy STR substrate binding sites under high-glucose conditions and high concentrations of glucose may be more potent and more effective than 3DG. But these hypotheses require further investigation.

Under basal conditions, our data showed a stimulatory effect of an acute administration of 3DG on GLP-1 secretion. Furthermore, acute exposure of STC-1 cells to 3DG upregulated STR signaling, thereby elevating intracellular Ca^2+^ concentration. Moreover, inhibition of STRs with lactisole decreased GLP-1 secretion triggered by 3DG under basal conditions. Together with the results observed under glucose-free conditions, STR was involved in 3DG-induced basal GLP-1 secretion. Surprisingly, inhibition of SGLT1 with phloridzin also reduced GLP-1 secretion in the presence of 3DG and 5.6 mM glucose (Supplemental [Supplementary-material supplementary-material-1]). It has been found that phloridzin could abolish GLP-1 secretion triggered by low concentrations of glucose (0.5 mM) in GLUTag cells [7], which indicated the significance of electrogenic action of SGLT1 in mediating GLP-1 secretion triggered by low concentrations of glucose in enteroendocrine L-cells. Thus, the decreased GLP-1 secretion should be attributed to the inhibitory effect of phloridzin on low-glucose-induced GLP-1 secretion. Collectively, under basal conditions, 3DG is likely to activate STR and thereby synergizes with low glucose to amplify GLP-1 secretion from STC-1 cells. Although STR activation also activates adenylate cyclase and the formation of cAMP [26], acute exposure to 3DG had any effect on intracellular cAMP in the presence of free glucose and low glucose. It is possible that STR activated by a variety of compounds with diverse chemical structures generates distinct patterns of intracellular signals [30].

In the present study, we found that a single oral administration of 3DG increases basal plasma GLP-1 levels *in vivo*. This effect could be attributed to the low absorption rate of individual dietary 3DG [16, 20]. The low absorption rate may help luminal 3DG to be exposed to a greater length of the gut where GLP-1-producing L-cells are present. In the present study, increased duodenum 3DG levels in 3DG-treated rats were observed. It is well documented that STRs are expressed in enteroendocrine L-cells along the gastrointestinal tract [31]. STR expression level in intestinal tissues in 3DG-treated rats was also detected. Consistent with the expression data in 3DG-treated STC-1 cells under basal conditions, TAS1R2 and TAS1R3 were upregulated in the duodenum of 3DG-treated rats. Thus, it is possible that the upregulation of STRs is involved in 3DG-induced basal GLP-1 secretion in rats in combination with the findings shown under glucose-free and 5.6 mM glucose conditions *in vitro*. As acute 3DG administration has a stimulatory effect on basal GLP-1 secretion, whether 3DG preload could minimize postprandial glycemic excursions, liking 3-*O*-methylglucose [32]. However, regarding the effect of continuous exposure to 3DG, it was shown to have a deleterious effect on glucose homeostasis [16, 18], liking glucose. Furthermore, a 2-week administration of exogenous 3DG decreased basal GLP-1 secretion [16]. It is also likely that the acute basal GLP-1 secretion is associated with inherent defense function to inhibit energy intake.

## 5. Conclusion

In summary, this study first presents the evidence that, under glucose-free conditions, acute exposure to 3DG directly stimulates GLP-1 secretion and that, under basal conditions, 3DG synergizes with low glucose to potentiate GLP-1 secretion. Although the molecular mechanisms remain unclear, our study has shown that 3DG-induced acute basal GLP-1 secretion is partly due to STR activation. These findings help us to gain a better understanding of the role of 3DG in health and disease.

## Data Availability

The data used to support the findings of this study are included within the article.

## Supplementary Materials

Supplementary Materials Supplementary Figure 1: effects of SGLT1 inhibitor phloridzin and GLUT2 inhibitor phloretin on GLP-1 secretion in STC-1 cells under conditions of 300 ng/ml and 5.6 mM glucose. The results shown are representative of three independent experiments and are expressed as the mean ± SD. The data for GLP-1 levels are expressed as a percent of the untreated group. ^*∗*^*P* < 0.05, ^#^*P* < 0.05.
