# Supplementary material for: 3-Deoxyglucosone Induces Glucagon-Like Peptide-1 Secretion from STC-1 Cells via Upregulating Sweet Taste Receptor Expression under Basal Conditions
Source: Int J Endocrinol. 2019 Oct 23;2019:4959646. doi: 10.1155/2019/4959646 (PMC6854250; doi:10.1155/2019/4959646)
Supplement: Supplementary Materials — Supplementary Figure 1: effects of SGLT1 inhibitor phloridzin and GLUT2 inhibitor phloretin on GLP-1 secretion in STC-1 cells under conditions of 300 ng/ml and 5.6 mM glucose. The results shown are representative of three independent experiments and are expressed as the mean ± SD. The data for GLP-1 levels are expressed as a percent of the untreated group. ∗P < 0.05, #P < 0.05. [file 4959646.f1.docx]

**Supplementary material**


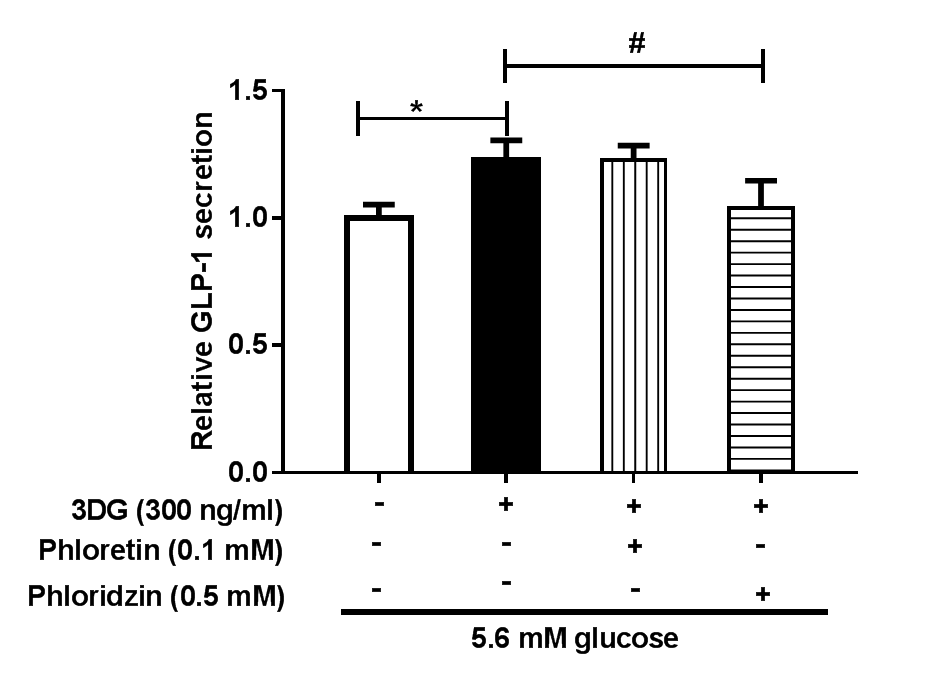


**Supplementary Figure 1. Effects of SGLT1 inhibitor phloridzin and GLUT2 inhibitor phloretin on GLP-1 secretion in STC-1 cells under conditions of 300 ng/ml and 5.6 mM glucose.** STC-1 cells were incubated for 1 h with either 0 or 300 ng/ml in the presence and absence of phloretin (0.1 mM) and phloridzin (0.5 mM) under 5.6 mM glucose conditions. The GLP-1 concentration in the supernatant was measured by ELISA. The data were expressed as a percent of the untreated group. ^*^*P*<0.05, ^#^*P*<0.05 (n = 6).
